# Pain drawings in somatoform-functional pain

**DOI:** 10.1186/1471-2474-13-257

**Published:** 2012-12-20

**Authors:** Niklaus Egloff, Rafael JA Cámara, Roland von Känel, Nicole Klingler, Elizabeth Marti, Marie-Louise Gander Ferrari

**Affiliations:** 1 Division of Psychosomatic Medicine, Department of General Internal Medicine, Inselspital, Bern University Hospital and University of Bern, Bern, Switzerland; 2 Department of General Internal Medicine and Department of Orthopedic Surgery, Bern University Hospital and University of Bern, CH-3010, Bern, Switzerland

**Keywords:** Chronic pain, Functional pain syndromes, Pain drawing, Somatoform pain disorder

## Abstract

**Background:**

Pain drawings are a diagnostic adjunct to history taking, clinical examinations, and biomedical tests in evaluating pain. We hypothesized that somatoform-functional pain, is mirrored in distinctive graphic patterns of pain drawings. Our aim was to identify the most sensitive and specific graphic criteria as a tool to help identifying somatoform-functional pain.

**Methods:**

We compared 62 patients with somatoform-functional pain with a control group of 49 patients with somatic-nociceptive pain type. All patients were asked to mark their pain on a pre-printed body diagram. An investigator, blinded with regard to the patients’ diagnoses, analyzed the drawings according to a set of numeric or binary criteria.

**Results:**

We identified 13 drawing criteria pointing with significance to a somatoform-functional pain disorder (all p-values ≤ 0.001). The most specific and most sensitive criteria combination for detecting somatoform-functional pain included the total *number of marks*, the *length of the longest mark,* and the presence of *symmetric patterns*. The area under the ROC-curve was 96.3% for this criteria combination.

**Conclusion:**

Pain drawings are an easy-to-administer supplementary technique which helps to identify somatoform-functional pain in comparison to somatic-nociceptive pain.

## Background

Pain drawings (PDs) are widely used to record subjective pain symptoms. In addition to good history taking, physical examinations, and results of biomedical tests, they can support to differentiate several types of local pain syndromes, such as chronic low back pain [1,2], chronic shoulder pain [3], neurogenic pain [4], and headaches [5].

Instead of focusing on any particular anatomical site, we concentrated on a particular *pain type*: We examined patients whose complaints could not be explained by either a peripheral structural or a neuropathic lesion. In psychiatric terms, the latter complaints are traditionally referred to as ‘*somatoform pain’,* whereas somatic medicine prefers the term *‘functional pain’ (*e.g. *functional pain syndromes)*. In the following, we will use the term *somatoform-functional pain* to describe this entire group of pain disorders.

Our aim was to identify those particular graphic criteria which specifically help to differentiate between somatoform-functional pain and somatic-nociceptive pain*.* More specifically, we wanted to find out which combination of features in a drawing has the best predictive value (sensitivity and specificity) to identify somatoform-functional pain. Based on the arguments of *Margolis* et al.*,* we strictly applied *quantitative* methods of picture analysis and avoided any qualitative or experience-based interpretations of pictures or signs [6].

## Methods

### Setting, design, and patients

We compared two groups of inpatients at a tertiary university hospital: one with *somatoform-functional* pain, the other with unequivocal *somatic-nociceptive pain*. We selected all patients solely according to these two pain types, but other basic characteristics were also noted.

Patients with *somatoform-functional* pain were recruited from the *medical psychosomatic* department. The defining eligibility criterion was a pain without an explanatory morphologic correlate. Such a correlate was excluded by standard clinical, serological and radiological methods.

Patients with *somatic-nociceptive* pain were recruited from the *orthopedic* department. Only patients with a clear peripheral correlate, verified by standard clinical and technical diagnostic methods (i.e. X-ray, MRI or CT), were included from this department. Patients with pain of neuropathic origin (e.g. diabetic neuropathy, spinal canal stenosis, discopathia), cancer-related pain and those with inflammatory pain (e.g., rheumatoid arthritis) were not included in this pilot study.

In both departments the treating specialists made diagnoses according to the usual Western standards and diagnostic classification systems (ICD-10, DSM-IV).

To assess depressive mood we used the depression subscale of the Hospital Anxiety and Depression Scale (HADS-D) [7,8]. To limit potential confounding of somatic-nociceptive pain by overt psychological factors, we excluded patients from the orthopedic department who endorsed a comorbid psychiatric disorder.

The patients were individually instructed by the investigator to mark all painful body areas with a red pencil on a letter-sized body diagram. The used body diagram shows four views of the whole body: frontal view, rear view, right and left lateral views (size: 105 mm from neck to sole). Additionally, there are enlarged views of the head, the neck, and the distal extremities. Patients were not given any instructions on *how* to apply the pencil (e.g. hatched or solidly filled areas), nor were they advised about the use of particular signs (e.g. circles, crosses, or arrows).

The study protocol has been approved by the local ethical committee and all patients provided written informed consent.

### Definition of pain drawing criteria

The evaluation of all PDs was carried out by only one investigator. He was blinded with regard to the pain diagnosis and the department’s origin of the patients. The inventory of the drawings is based on their graphic features only. We strictly renounced any interpretive criteria (e.g. “not corresponding to anatomic structures”). The criteria included objective aspects only, e.g. the *form* (lines, hatches, circles, and rectangles) and the *orientation* (e.g. horizontal lines, symmetrical distribution). We took the *position with regard to the template border* (i.e. lines following the contour of the body scheme, or marks exceeding the border of the body scheme) into account. In addition, we considered *quantitative criteria* (i.e. number of marked quadrants, number of pain regions, and number of different marks) as well as the *size* of the marks (i.e. longest mark). Overall, our evaluation included a comprehensive panel of 24 graphic criteria (cf. below).

### Data analysis

We compared the pain groups in terms of health characteristics and pain drawing marks. In the latter comparison we distinguished between *frequent* marks, occurring in at least 25% of the patients in one of the pain groups, and *rare* marks. The frequency of category-type statements was expressed in percentages, whereas the mean ± standard deviation (SD) was used for continuous variables. P-values for differences of PDs in the two pain groups were computed by Mann–Whitney *U* test and Pearson chi-square test or Fisher’s exact test where appropriate. Significance level was set as 0.001 with Bonferroni corrections for 24 comparisons. Furthermore, two multivariate logistic regression analyses were performed for *frequent* marks with respect to control variables: One basic model (analysis 1) adjusted only for *age* and *gender,* a second (analysis 2) additionally for *other characteristic group differences*. Principally, all covariates were tested which might co-influence the drawings of the two different pain groups (e.g. age, gender, pain duration, pain severity, as well as mood). Ninety-five % confidence intervals were calculated for all odds ratios.

Finally, in order to identify the combination of criteria with the highest selectivity (i.e. sensitivity and specificity), we used receiver operating characteristic (ROC) curves: In a first step, we identified the criterion with the highest selectivity out of all significant frequent criteria. In a second step, we identified those criteria which added the most to the selectivity of the first criterion. To achieve this, the first criterion was separately modeled (i.e. two variables per model) with each of the other frequent criteria by binary logistic regression. Pairs of criteria which both contributed to the model were combined with the natural logarithm (ln) of the equation of the model. For instance, if the odds ratio was 2 for criterion A and 3 for criterion B, the score would be ln2 times criterion A plus ln3 times criterion B. This method allows to quantify the additive predictive value of multiple criteria. The most sensitive and specific *combination* was again analyzed by ROC-curve analysis. The two best matching criteria of this second combination were afterwards modeled once more by binary logistic regression, this time with a third criterion, in order to finally identify the most appropriate triple-combination.

## Results

### Description of the patients

**Figure 1:**
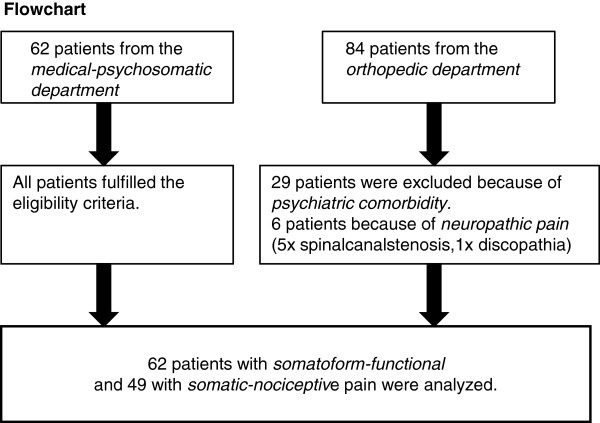
**Flow-chart of patients selected for analysis.** This figure illustrates the recruitment process

The flowchart (Figure 1) describes the selection of the 62 (56%) patients with somatoform-functional pain and the 49 (44%) patients with somatic-nociceptive pain. Of the orthopedic group, 6 patients were excluded because of neuropathic pain syndrome and 29 because of psychiatric comorbidity.

Among the patients from the *medical-psychosomatic* department, chronic *functional somatic syndromes* included fibromyalgia (n = 14), chronic tension headache (n = 4), chronic temporomandibular pain (n = 1), atypical facial pain (n = 1), chronic back pain (n = 16), functional abdominal pain or chronic pelvic pain (n = 8). Other patients suffered from inexplicable pain of the trunk (n = 8), pain-related functional hemi-syndromes (n = 6), inexplicable chronic postsurgical pain (n = 1), chronic shoulder/arm pain (6), and chronic cervico/thoracic pain (n = 11).

Among the patients from the *orthopedic* department, acute traumatic pain included traumas of the upper extremities or shoulders (n = 11), bone fractures or joint lesions of the lower extremities (n = 15), thoracic, vertebral or pelvic traumas (n = 7). Degenerative osteoarthritic pain included hip arthritis (n = 9), knee arthritis (n = 9), degenerative shoulder pain (n = 5), and degenerative low back pain (n = 7).

Sixteen patients with somatoform-functional and 13 patients with somatic-nociceptive pain had more than one diagnosis for their pain type.

Further patient characteristics are shown in Table 1 together with p-values for group differences.

**Table 1:** Health characteristics of 111 pain patients according to diagnostic groups

**Variable**	**Somatoform-functional pain (n = 62)**	**Somatic-nociceptive pain (n = 49)**	**P-value**
Age (years)	47±12 (20–75)	55±17 (16–84)	0.005
Female sex (%)	62.9	34.7	0.003
Pain duration (months)	99±115 (3–451)	17±24 (0.1-100)	<0.001
Pain level (NRS)	6.4±2.2 (1–10)	3.5±1.4 (1–7)	<0.001
Depressive disorder (%)	87.1	0	<0.001
Anxiety disorder (%)^1)^	35.5	0	<0.001
Opioids (%) (includes Tramadol)	30.2	6.1	<0.001
NSAIDs (%) (includes Paracetamol and Metamizol)	50.0	93.3	0.001
Antidepressants (%)	85.5	0	<0.001
Antiepileptics (%)	24.2	0	<0.001

1) includes PTSD.

*NSAIDs* = non-steroidal anti-inflammatory drugs; *NRS* = numerical rating scale.

Data are given as means ± SD with range in parentheses or percentage values. Analyses used Mann–Whitney *U* test and Pearson chi-square test or Fisher’s exact test (where appropriate).

### Description of pain drawings

Thirteen of the 24 analyzed graphic criteria displayed differences of the p-values ≤ 0.001. Eleven of these criteria were *frequent* (= occurred >25% in one of the two pain groups), cf. Table 2.

**Table 2:** Pain drawing criteria according to diagnostic groups

**Frequently**^1)^**occuring drawings marks**			
**Pain drawing criteria**	**Somatoform-functional pain (n = 62)**	**Somatic-nociceptive pain (n = 49)**	**P-value**
1. Number of marked pain regions^2)^	9.7±7.4 (1–33)	1.6±1.0 (1–6)	<0.001
2. Total number of marks	13.5±11.8 (1–73)	2.1±1.4 (1–8)	<0.001
3. Number of different types of marks	2.6±1.3 (1–6)	1.3±0.6 (1–3)	<0.001
4. Number of affected quadrants	3.0±1.1 (1–4)	1.6±0.9 (1–4)	<0.001
5. Number of symmetric marks	6.9±7.5 (0–30)	0.4±0.9 (0–4)	<0.001
6. Number of “over the border” marks	1.4±2.5 (0–13)	0.4±0.9 (0–5)	0.010
7. Length of the longest mark, in mm	37.2±26.0 (2–105)	14.7±9.2 (3–40)	<0.001
8. Symmetric patterns yes, %	75.8	16.3	<0.001
9. Neck involved yes, %	74.2	4.1	<0.001
10. Circle mark yes, %	16.1	26.5	0.179
11. Point mark yes, %	37.1	4.1	<0.001
12. Long lines yes^3)^, %	62.9	6.1	<0.001
13. Strict horizontal mark yes, %	25.8	2.0	<0.001
14. Hatching mark yes, %	38.7	14.3	0.004
**Rarely**^1)^**occurring drawing mark**			
15. Half-side pattern yes, %	9.7	0	0.033
16. Sternocleidomastoid involved yes, %	21.0	2.0	0.003
17. Periorbital pain yes, %	11.3	0	0.017
18. X marks yes, %	8.1	22.4	0.054
19. Potato mark yes, %	24.2	18.4	0.459
20. Right angle yes, %	14.5	0	0.004
21. Contour pain yes, %	19.4	0	0.001
22. Towing bar yes, %	8.1	0	0.065
23. Radiation hand yes, %	19.4	0	0.001
24. Ear crest yes,%	11.3	0	0.017

1) Marks referred to as “frequently occurring“ appeared in ≥ 25% of cases in one of the two groups. Marks referred to as “rarely occurring“ appeared in less than 25% of cases in both groups.

2) If the same pain region was repeatedly marked in the different views of the body diagram, it was counted once only under this item.

3) A mark was considered to be “long” if it exceeded the length of a forearm (≥ 18 mm) in the template.

In the first multivariate analysis, Analysis 1 adjusted for *age* and *gende*r, 7 of the frequent criteria had an OR > 3 (range 3.72–53.95). In the second multivariate analysis, Analysis 2 adjusted for *age*, *gender, pain duration, pain severity* and *mood*, 10 criteria had an OR > 3 (range 3.49–140.4), cf. Table 3.

**Table 3:** **Odds ratio of*Frequent*drawing criteria in somatoform-functional vs. somatic-nociceptive pain on logistic regression**

**Pain drawing criteria**	**Analysis 1**		**Analysis 2**	
	**Adjusted for age and gender**		**Adjusted for age, gender, pain duration, pain severity and mood**	
	**OR**	**95% CI**	**OR**	**95% CI**
1. Number of marked pain regions	2.39	1.59-3.60	**4.64**	1.64-13.1
2. Total number of marks	1.84	1.37-2.48	**4.42**	1.61-12.13
3. Number of different types of marks	**3.93**	2.08-7.44	**3.65**	1.27-10.54
4. Number of affected quadrants	**3.74**	2.22-6.27	**3.97**	1.62-9.72
5. Number of symmetric marks	2.66	1.61-4.40	**3.49**	1.48-8.26
6. Number of “over the border” marks	1.50	1.03-2.18	1.72	0.95-3.10
7. Length of the longest mark, mm	1.57	1.25-1.98	1.52	1.07-2.16
8. Symmetric patterns yes, %	**19.6**	6.46-59.38	**21.94**	3.59-134.11
9. Neck involved yes, %	**53.95**	11.29-257.78	**140.4**	7.80-2527.29
10. Circle mark yes, %	0.56	0.21-1.55	0.31	0.05-1.77
11. Point mark yes, %	**12.32**	2.55-59.53	**9.46**	0.76-118.02
12. Long lines yes, %	**22.99**	6.12-86.39	**16.94**	2.22-129.38
13. Strict horizontal lines yes, %	0.074	0.01-0.62	0.08	0.00-1.71
14. Hatching mark yes, %	**3.72**	1.33-10.42	**8.66**	1.56-48.14

Explanations for models adjusted for age and gender:

For *categorical* variables (yes, no) odds ratios indicate the following: if e.g. criterion no.11 (*point mark*) was present, the odds of a patient suffering from somatoform pain was 12-fold higher than suffering from somatic pain.

For *continuous* variables odds ratios indicate the following: e.g. for one additional mark (e.g. criterion no. 2) the odds of the patient suffering from somatoform pain as opposed to somatic pain was increased by factor 1.84.

Criterion no.7 (*longest mark*) was measured in 5 mm steps: For every increase of the length of the longest mark by 5 mm, the odds of the patient suffering from somatoform as opposed to somatic pain was increased by factor 1.57.

Bold figures: pain drawing criteria with OR > 3.

**Figure 2:**
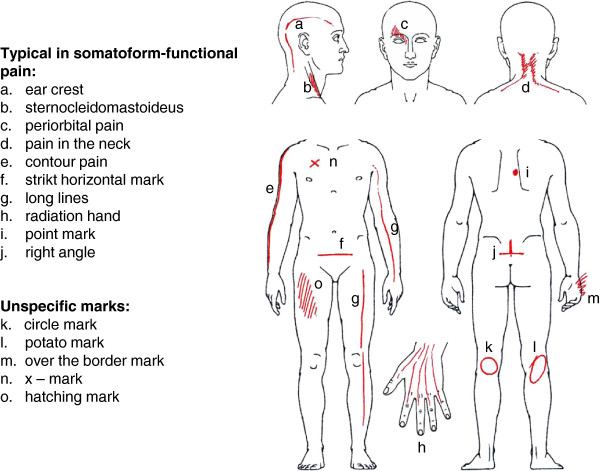
**Typical drawing marks.** Index of the discussed drawing marks

**Figure 3:**
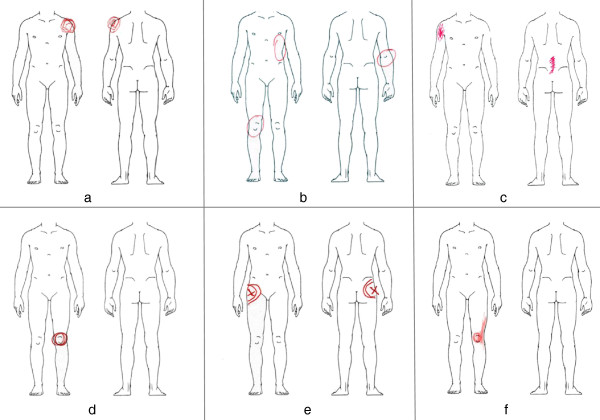
**Typical PDs from patients with somatic-nociceptive pain.** Pictures **a-c** show orthopedic trauma pain. Pictures **d-f** are examples for degenerative pain caused by arthralgia. Typically, somatic-nociceptive pain is well localised

**Figure 4:**
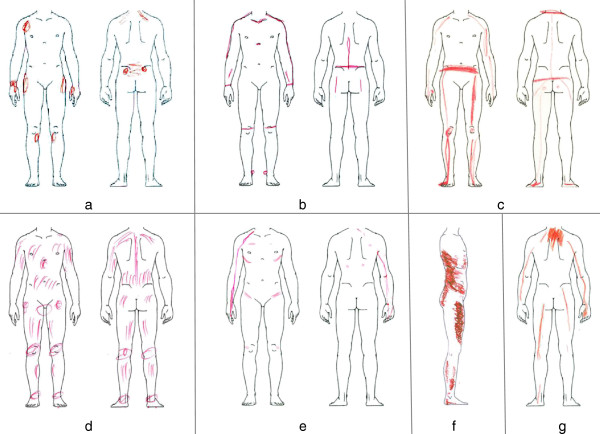
**Typical PDs from patients with somatoform-functional pain.** Picture **a-g** show pain drawings of patients with **a-g** medically inexplicable pain syndromes. Somatoform-functional pain is typically associated with symmetric patterns, long lines, and a higher number of marks

Figure 2 gives a graphic index of the investigated PD marks. A selection of representative examples of somatic-nociceptive and somatoform-functional PDs is illustrated with Figures 3 and 4.

### Most sensitive and specific combination

**Figure 5:**
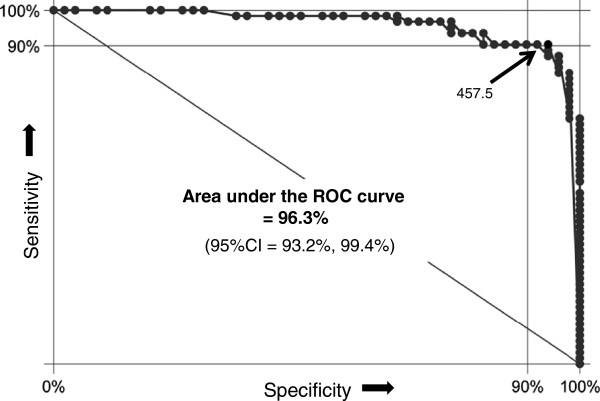
**ROC-curve of the best pain-drawing criteria group.** This receiver operating characteristic (ROC) curve illustrates the sensitivity and specificity of the combination of pain-drawing criteria with the highest diagnostic accuracy (*total number of marks*, *length of the longest mark*, and *presence of symmetric pain zones*). Virtually each possible cut-point is plotted against specificity (x axis) and sensitivity (y axis). The cut-off of 457.5 generated 93.9% specificity and 90.3% sensitivity, which represents the mathematical optimum of correctly diagnosed cases

The most equilibrated *criteria combination* to differentiate between the two types of pain included the *total number of marks*, the *length* of the longest mark, and presence versus absence of *symmetric patterns* (statistic calculations are shown in Tables 4 and 5). This means that PDs with a *greater number* of marks, typically with *symmetric patterns* and *long* marks, are most likely of somatoform-functional origin. The area under the ROC-curve for this triple criteria combination was 96.3% (Figure 5).

**Table 4:** Binary logistic regression of the most sensitive and specific combination of pain drawing criteria

**Pain drawing criteria**	**OR/unit (95% CI)**	**Log likelihood**	**p-value**
Total number of marks	1.7 (1.3, 2.3)	- 25	< 0.001
Length of the longest mark	1.1 (1.0, 1.1)		0.029
Symmetric patterns (yes/no)	6.5 (1.6, 26.9)		0.010

This tabulated group lists the three best pain drawing criteria (two continuous plus one binary) with the highest power to distinguish somatoform-functional from somatic-nociceptive pain, the criteria are included in a model as separate variables.

**Table 5:** ROC-formula of the best combination of pain drawing criteria

**Formula to compute the score**	**Mean ± SD (Range)**	**OR/SD (95% CI)**	**Log likelihood**	**p-value**
55 points multiplied with the total number of marks plus	748 ± 700 (81 4397)	1114 (62, 20’132)	- 25	- 0.001
32.2 points multiplied with the length of the longest mark plus				
187 points if symmetric patterns are present				

This table shows the properties of the best pain drawing criteria after combining them to one single continuous variable. The combination to one variable is necessary to obtain one single receiver operating characteristic curve including the diagnostic properties of all three criteria.

Furthermore, the effective predicting score value of an individual drawing could be computed by using the following score formula: *55* × *the total number of marks*, plus *32.5* × *for each five-mm of the longest mark*, plus an additional amount of *187* if *symmetric patterns* are present. For instance, a pain drawing with 8 marks, in which the extension of the longest mark is 25 mm, including symmetric patterns, yields a score value of 788. With a score of ≥ 779.5, the positive predictive value for a somatoform-functional pain would be nearly 100% (95%CI: 91.8%, 100%). Sixty-nine percent of our patients with somatoform-functional pain scored ≥ 779.5 (69.3%, 95%CI: 56.3%, 80.4%). The cut-off of 457.5 represents the mathematically optimal combination of *sensitivity* (90.3%; 95%CI: 80.1%, 96.4%) and *specificity* (93.9%; 95%CI: 83.1%, 98.7%) to discern somatoform-functional from somatic-nociceptive pain. This cut-off would still yield a positive predictive value of 94.9% for the probability of *somatoform-functional* pain (95%CI: 85.8%, 98.9%). Conversely, a score < 457.5 would generate a probability of *somatic-nociceptive* pain of 88.5% (95%CI: 76.6%, 95.6%).

## Discussion

This study is an approach to a better implementation of PDs as one tool in the diagnostic assessment of somatoform-functional pain syndromes. PDs of patients with *somatoform-functional* pain differ significantly with respect to a defined spectrum of graphic features. The frequency and the stereotypical appearance of these patterns procure a suitable tool for diagnostic purposes. The greatest power to identify somatoform-functional pain resulted in our study from the combination of the *number of marks*, *length of the longest mark*, and *presence* versus *absence of symmetric patterns*. The area under the ROC-curve for this triple criteria combination is more than 95% (Figure 5). Assuming that the percentage of our cohort of pain patients with somatoform-functional pain (56%) is representative for the pain population assessed in a tertiary pain center, 91.9% of these patients would be correctly classified by these three pain drawing criteria alone (95%CI: 85.2%, 96.2%). The prevalence of some drawing criteria was highly different between the two pain populations (e.g., neck involvement, symmetric patterns; Table 2). This difference mathematically explains why some of the odds ratios are unusually high (Table 3). Clearly, the absolute values of these odds ratios should not be overinterpreted but may underscore the observation that some of the pain drawing criteria are vastly different between the two groups of pain patients.

The two patient groups also differed vastly in terms of their *basic characteristics* (Table 1). This was expected because somatoform-functional pain shows known associations with for instance female gender and increased pain duration. In addition, more than 80% of our somatoform-functional pain patients suffered from clinical depression. It is generally known that somatoform-functional pain disorders are frequently associated with psychiatric disorders. Excluding patients with somatoform-functional pain with a concurrent psychiatric comorbidity would have yielded a clinically less representative sample. Nevertheless, their PDs should not be used as a *psychodiagnostic* test in disguise [9]. There are more useful tests available for specific psychodiagnostic screening purposes [10]. Our opinion is that the graphic patterns in somatoform-functional pain disorders illustrate several pain specific perceptional aspects. By definition, the indicated pain areas reflect, in contrast to the somatic-nociceptive pain, no injuries. The signs in somatorm-functional pain disorders may have diverse other causes. In the following, we will discuss some possible explanations illustrated with PDs.

Inexplicable functional pain syndromes are often associated with generalized hyperalgesia [11-13]. In such a case, even the perception of normal postural tone (Figure 4f), the force in muscle insertions (Figure 4a), or the weight exerted on joints (Figures 4b, 4c) is amplified and results in a painful sensation. This generalized hyperalgesia of the musculoskeletal system could also explain the high number as well as the symmetrical distribution of the patients’ complaints. Other graphic patterns are indicative of increased local muscle tension [14]. Obviously, the neck is an area of predilection for stress-associated muscular pain (Figure 2a, 2d): 75% of our patients suffering from somatoform-functional pain indicate in their PDs neck pain. Finally, another subtype of somatoform-functional pain can be found in patients suffering from severe posttraumatic stress disorder. Their initial physical pain related to the trauma often seems to leave an irreversible imprint on their body scheme which persists for years in some sort of “memory pain” [15,16] (Figure 4g: patient with whiplash-associated PTSD 10 years ago).

To conclude, we think that pain drawings of patients with somatoform-functional pain could hint at their cryptic and almost unknown pathophysiology. Generally, we recommend the PD-method as a helpful diagnostic adjunct in the assessment of any complex pain problems. Specially, the identification of patients with somatoform-functional pain is difficult and often related to frustrating diagnostic detours. An early identification of somatoform-functional pain is of clinical importance: Whereas treatment of somatic-nociceptive pain relies on analgesics (according to WHO criteria), the therapy of somatoform-functional pain considers dual antidepressants with analgesic properties alone or in combination with other modalities of multimodal pain treatment [17].

In this study we excluded orthopedic patients with a psychiatric comorbidity as well as pain patients with *both* somatoform-functional pain and somatic-nociceptive pain. When assessing PDs in clinical routine, however, one should keep in mind that the individual patient may also present with *combinations of different pain types*. Generally, we would not advise the use of PDs to rule out somatic-nociceptive pain, but recommend them as a *positive indicator* to identify somatoform-functional complaints.

We emphasize several limitations of our pilot study. Since we did not include any patients with neuropathic pain, tumor pain, or inflammatory rheumatic diseases, we cannot make any statement about the above mentioned criteria with regard to these patient groups. Because of more *multilocular* or *widespread* pain origins, these pain groups might show a closer overlap in their PDs with somatoform-functional pain syndromes than the classic orthopedic pain patients. For further differentiations more investigations are essential. Although we controlled for important demographic and clinical covariates, we are unable to exclude the possibility that factors other than the type of pain (e.g., the different clinical setting) might have contributed to differences in PD. Assessment of PD with only one rater does not allow to draw conclusions about the interrater reliability of PD.

## Conclusion

To sum up, PDs are an easy-to-administer technique helping the clinician to detect somatoform-functional pain in comparison with somatic-nociceptive orthopedic pain. Clinical experience shows that many patients do appreciate this personalized and documentative style as one means of the diagnostic approach.

## Abbrevations

(HADS-D): Hospital Anxiety and Depression Scale; (PDs): Pain drawings; (ROC): Receiver operating characteristic; (SD): Curves, standard deviation.

## Competing interests

The authors have no competing interests to report. All authors declare no financial or other relationships that might lead to a competing interest.

## Authors’ contributions

NE and MLG are responsible for the whole clinical concept and examinations, and RvK and RC for the study design and the statistical analyses. NE and RvK supervised the study on a medical-methodological level. NK, MLG and EM performed the clinical tests. All authors participated substantially in the acquisition or the analyses of the data. All co-authors were involved in drafting or revising the manuscript and have approved its final version.

## Prior presentations

All authors confirm that the manuscript has not been published elsewhere.

## Pre-publication history

The pre-publication history for this paper can be accessed here:

http://www.biomedcentral.com/1471-2474/13/257/prepub
